# Modelling and predicting forced migration

**DOI:** 10.1371/journal.pone.0284416

**Published:** 2023-04-13

**Authors:** Haodong Qi, Tuba Bircan

**Affiliations:** 1 Malmö Institute for Studies of Migration, Diversity and Welfare, Malmö University, Malmö, Sweden; 2 Stockholm University Demography Unit, Stockholm University, Stockholm, Sweden; 3 Department of Sociology, Vrije Universiteit Brussels, Brussels, Belgium; University of Macerata, ITALY

## Abstract

Migration models have evolved significantly during the last decade, most notably the so-called flow Fixed-Effects (FE) gravity models. Such models attempt to infer how human mobility may be driven by changing economy, geopolitics, and the environment among other things. They are also increasingly used for migration projections and forecasts. However, recent research shows that this class of models can neither explain, nor predict the temporal dynamics of human movement. This shortcoming is even more apparent in the context of forced migration, in which the processes and drivers tend to be heterogeneous and complex. In this article, we derived a Flow–Specific Temporal Gravity (FTG) model which, compared to the FE models, is theoretically similar (informed by the random utility framework), but empirically less restrictive. Using EUROSTAT data with climate, economic, and conflict indicators, we trained both models and compared their performances. The results suggest that the predictive power of these models is highly dependent on the length of training data. Specifically, as time-series migration data lengthens, FTG’s predictions can be increasingly accurate, whereas the FE model becomes less predictive.

## Introduction

The world is at a critical moment in the pursuit of fulfilling the Sustainable Development Goals—SDGs—by 2030 [1]; environmental crises, poverty, epidemics, and conflicts have been threatening decades of development gains. To adapt to these threats, millions of people have been forcibly displaced and in search for a safer and more livable place. Pressingly, many have lost their lives or gone missing along the journey [2]. To ensure safe and orderly migration, there is an urgent need to develop systems which can help anticipate where and when forcibly displaced people are likely to migrate [3, 4]. However, building such systems is extremely difficult, as the drivers and temporal dynamics of forced migration are inherently complex and the conventional models (in the sprite of gravity equation) are inadequate to explain and predict them [5]. To address this shortcoming, we propose a novel approach—a Flow–Specific Temporal Gravity (FTG) model, and examine the extent to which this approach may better explain and predict asylum-related migration, compared to conventional models.

Since its inception, the gravity equation has been the cornerstone for economists to examine the role of income maximization in shaping migration decisions (see e.g., [6–12]). It has also gained popularity in climatology, geography, earth observation, demography, political science and many other disciplines to study the impacts of climate, conflict, natural disasters and other shocks on human migration (see e.g. [13–19]). As panel data on global migration flows become more readily available [20], the techniques to estimate gravity models have evolved significantly, from simple cross-sectional estimation to more advanced Fixed-Effects (FE) models. The latter model class is designed to infer migration responses to not only spatial, but also temporal variations in time-varying predictors.

Although useful for inferring migration drivers, the theoretical foundation of the Fixed-Effects (FE) gravity models is not unequivocal. In particular, these models assumed that all migrants are forward–looking utility maximizers who move voluntarily and spontaneously for better opportunities and livelihoods elsewhere [21]. This assumption, however, has struggled to conform to the phenomenon of involuntary immobility, namely a larger share of people does not migrate despite income and opportunity gaps [22–24]. Empirically, the FE models assumed homogeneous effects of predictors, which tend to yield large and significant estimates. However, recent study showed that, when such models are stratified by dyad flows, the parameter estimates exhibit pervasive heterogeneity across contexts [25]. This suggests that cross-border migration as responses to economic changes as well as to climate/environmental, geopolitical, among other shocks are *not* uniformly spontaneous.

To address the above-mentioned shortcomings, we derived a Flow–Specific Temporal gravity (FTG) model which has a less restrictive parameterization, compared to the traditional FE models. Specifically, the model exploits only temporal, but not spatial, variation in migration panel data. This feature allows us to study how migration drivers may vary across flows. It also allows us to exclusively infer the temporal dynamics of forced migration (by isolating the spatial variation in the panel data).

Using EUROSTAT data and climate, economic, and conflict indicators gathered from various data sources, we showed that the performances of FTG and FE models are highly dependent on the length of training data. In particular, as time-series migration data lengthens, the FTG model can make increasingly accurate predictions, whereas the FE model becomes less predictive. Nevertheless, it is important to stress that the FTG model is “Data Hungry”, which implies that its adoption is likely to be slow as most time-series migration data are still short and need time to grow longer.

## Forced migration: Processes and drivers

Migration journeys can be highly nonlinear as they are often the product of entangled and sequential decision making process: from an initial intention to a decision, and ultimately to a realized movement [26–29]. For those who move voluntarily, e.g., for work, family, and study reasons, migration may proceed in a planned manner. However, for forcibly displaced people, the decision to flee might not be intended initially. Hence, the process of forced migration may entail multiple stages. For example, in the event of a crisis, the affected population might be internally displaced initially. Later, some might cross administrative areas or move abroad, and thus become forced migrants, while others return home. Moreover, the onward journey following initial displacement might be particularly challenging, as the regular migration channels are unlikely to be accessible and affordable [30, 31].

**Fig 1:**
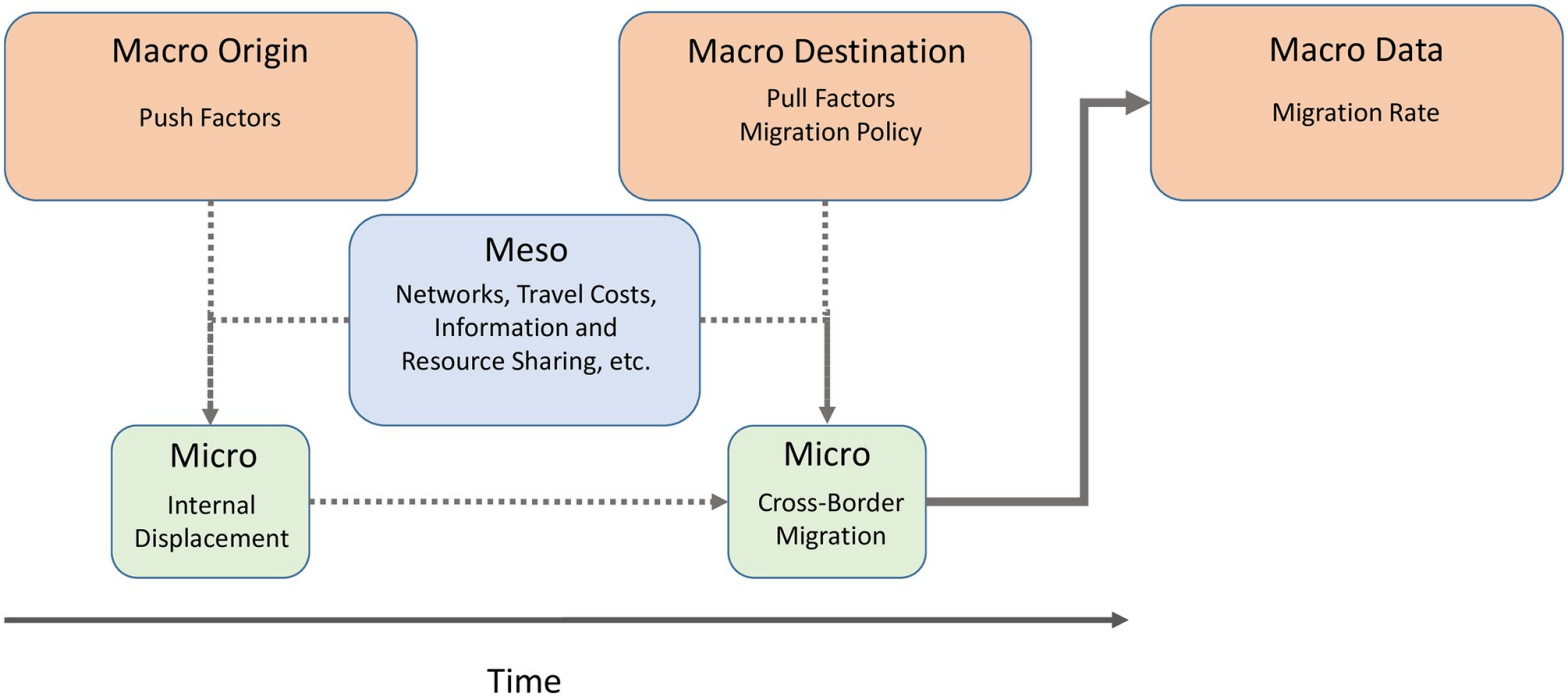
Forced migration process, drivers, and data source: Authors’ adaption based on previous studies [32–34].

To illuminate the lengthy process of forced migration, we conceptualize a framework in Fig 1 which is adapted from previous studies [32–34]. A key feature in this framework is that we distinguish between the initial displacement and the later cross-border migration. The former is triggered by a macro shock, hence is unplanned, whereas the latter involves searching for a destination and planning for the move. Another important note in Fig 1 is the temporal dimension between the two stages. While those who move voluntarily might have resources ready for departure once an opportunity emerges, the circumstances for forcibly displaced people are likely to be different. Resources and wealth might be wiped out during a catastrophic conflict or natural disaster. This might have an impact on people’s perceptions and on the opportunity structures and hence on their aspirations and capabilities [21, 35]. Restoring lost capability requires time. Hence, the temporal dimension is critical to understand the process of forced migration.

The mechanisms underlying migratory process can be highly complex and contextually contingent. In the case of forced migration, the drivers may evolve overtime. For example, the initial displacement could be triggered by (hard) push factors such as armed conflicts, extreme weather conditions, economic crises, famine, among other abrupt events [33, 36]. The onward migration decision following displacement might be shaped by pull factors (associated with the destination area), intervening obstacles, and individual factors [32].

The conflict–migration nexus has existed long before the constitution of the 1951 Refugee Convention [37]. Theoretically, the threat–based decision model postulated that perceived (unbearable) threat to personal security is the main driver for people to migrate away from a conflict zone [38–40]. Empirical studies have reported high rates of out–migration during armed conflict, civil war, genocide and human right violations [39, 41].

Recent studies have investigated the relation between extreme weather events and migration [42]. Empirical findings suggest that droughts and floods as well as temperature fluctuations can act as push factors for out–migration [13, 18, 43, 44]. However, the causal pathways between environment/climate conditions and migration can be complex [14, 19, 45, 46]. This is because extreme weather conditions can increase the risks of conflicts and economic hardships, which could increase emigration rates in an indirect way [13, 47, 48].

While the theories and empirical evidence mentioned above generally imply an immediate out–migration response to different geopolitical and climatic shocks, they are silent about what happens after initial displacement, namely where people move to and how the location choices are made. In our framework, we assume that the second stage of decision making involves a search process for potential destinations, and evaluations of the benefits and costs of moving to these destinations. This step is similar to solving an income maximization problem postulated by the classic economic theory of migration [6], which is, however, challenged by two empirical facts. First, the stock of international migrants merely accounts 3.5% of the world population [49]. This figure is way below what one would expect if every single working age individual optimizes their income through international migration. Hence, the economic approaches have struggled to explain why most people do not migrate despite income and opportunity gaps [22–24]. Second, those who move, particularly to the OECD countries, tend to be positively selected on education, and actually receive a lower return on their skills [9].

Economic theories of international migration have evolved considerably overtime with elements added on to the simple income maximization problem, such as the welfare magnets [50], income risks and inequality [51, 52], credit constraints [10–12], and multilateral resistance to migration [7, 8], among others. While these theories may seem to be applicable to the onward migration decision after initial displacement, a key question is whether a displaced person may have the same capabilities as an economic migrant, namely the resources needed in order to move to a place most likely to match their aspirations [22, 53, 54].

The decision to migrate is likely to be an outcome of an individual’s interactions with macro– and meso–level factors [55]. One example of such interactions is social navigation, which tends to have a strong temporal pattern when circumstances are dynamic, uncertain, and insecure [56, 57]. The temporal aspect is therefore not limited to the restoration of individual’s capability, it may also emerge from changing macro conditions in both origin and destination countries, as well as from the interaction with meso–level factors. As shown in Fig 1, migration policies in different destination countries might shift overtime. If they become more restrictive, migration costs will rise, which may, in turn, prolong the time needed to restore capabilities. On the other hand, meso–level factors, such as migration networks, may help facilitate information flows and establish migration channels between the origin and destinations. It may also help ease credit constraints. These factors (albeit often unobservable) could potentially lower the migration costs, and hence accelerate the migratory process.

## Data

The empirical analysis in this article relies on various data sources. For the flows of forced migration, we make use of EUROSTAT asylum statistics. To derive indicators for push and pull factors (as depicted in Fig 1), we use data from the World Bank, Standardised Precipitation–Evapotranspiration Index (SPEI), and Uppsala Conflict Data Program (UCDP).

### Forced migration data

EUROSTAT routinely compiles information on the number of first–time asylum applications lodged in different EU countries. We combine this information with population data from the world bank to compute the asylum-seeking rate (hereafter ASR). That is, the number of first–time asylum seekers per 100 people remained in a given country of origin.

**Fig 2:**
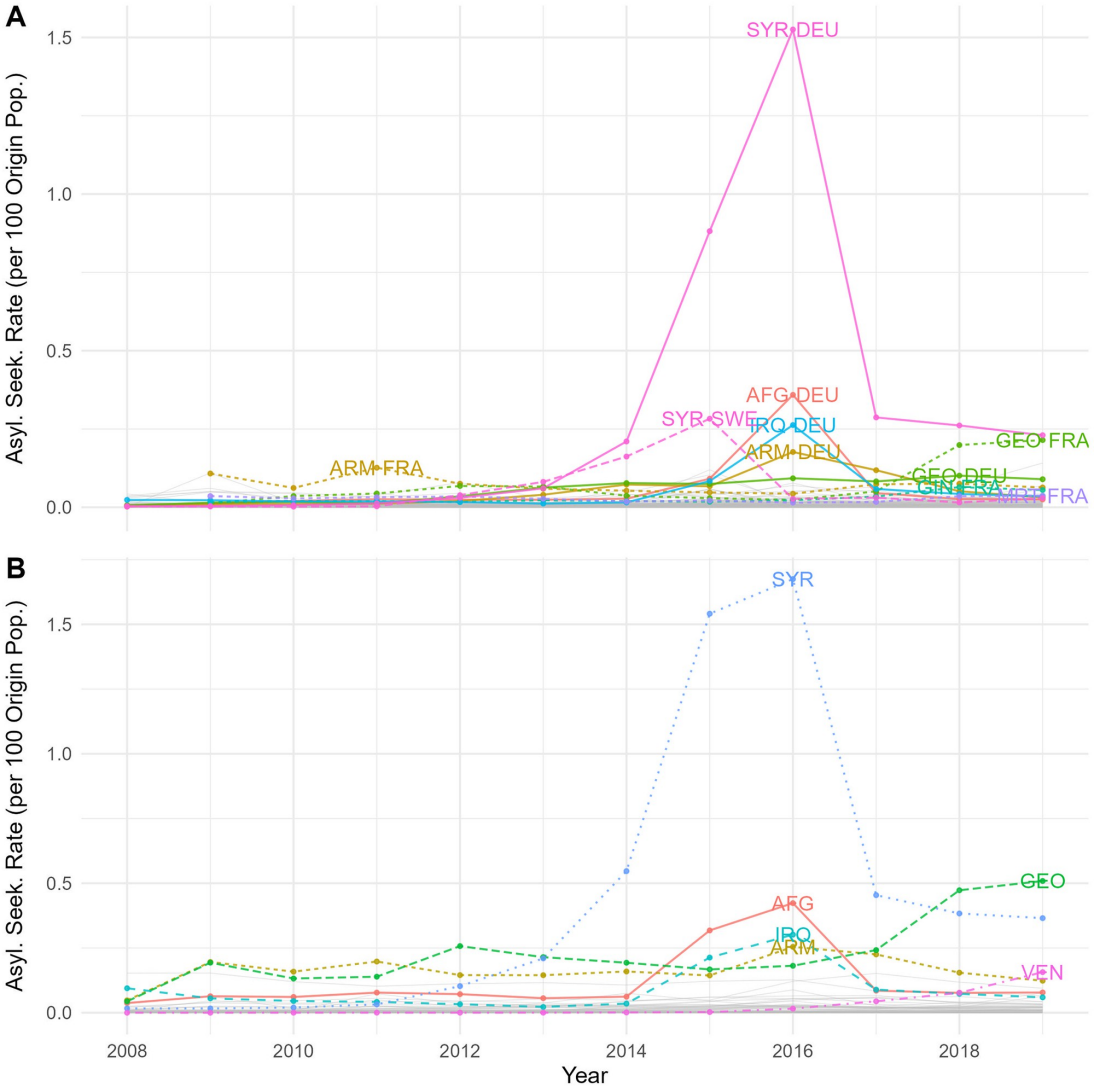
Asylum seekers (per 100 origin population). A: Asylum-seeking rate by origin–destination dyad flows. B: Asylum-seeking rate by countries of origin.

Fig 2 depicts the ASR by origin–destination dyad flow (Panel A). It is evident that Germany was the most common destination for asylum seekers; during the so-called “Refugee Crisis”, about 2.5% of the population in Syria fled to Germany. In Panel B of Fig 2, we sum up flow-specific ASR by origin countries. The patterns show that, in total, 3.2% of the population in Syria fled to the EU in 2015-2016 and the corresponding figures in Afghanistan, Iraq, and Armenia are 0.7%, 0.5%, and 0.4%, respectively.

### Conflict indicator

**Fig 3:**
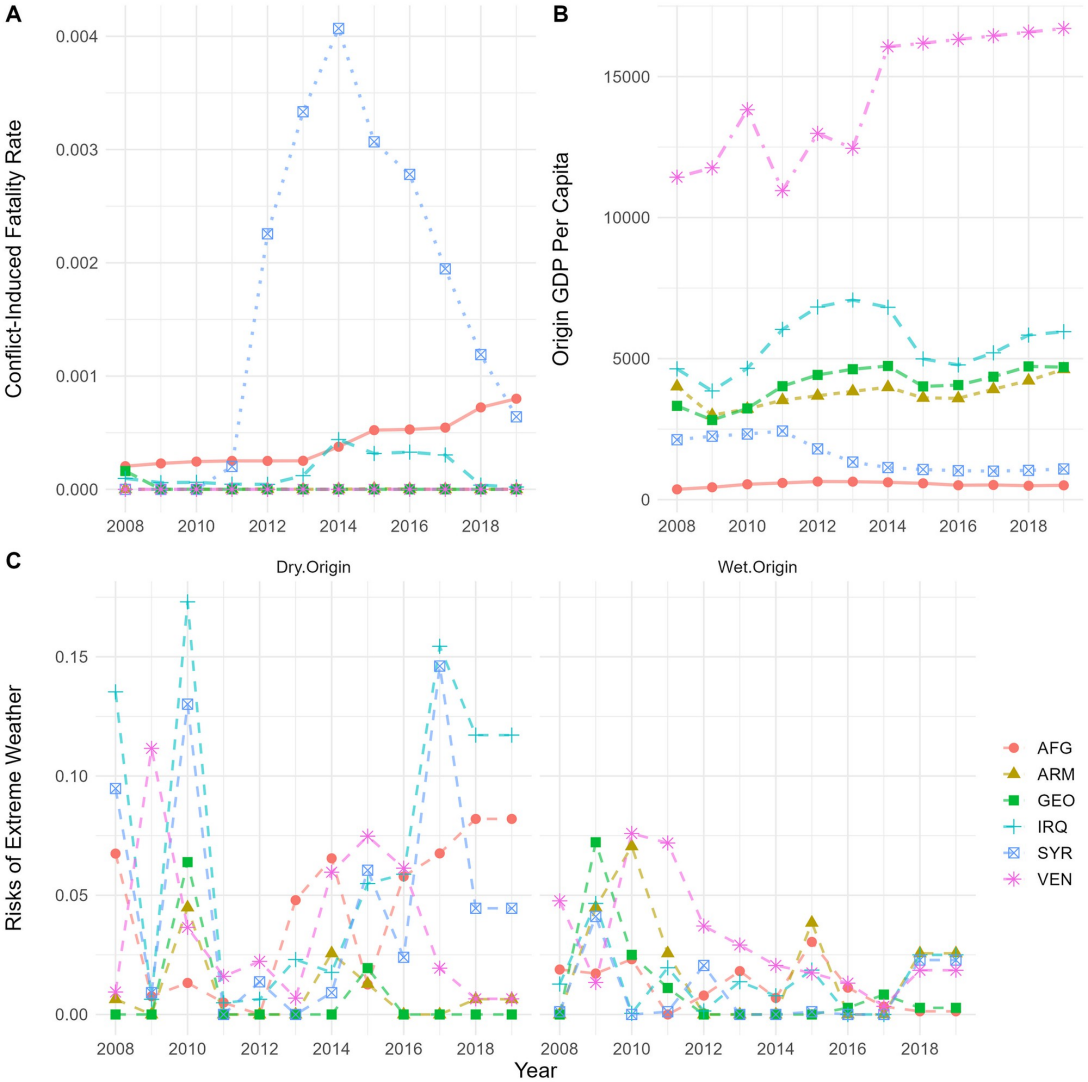
Conflict, economic, and climate data. A: Trends in conflict–induced mortality rates. B: Trends in GDP per capita. C: Trends in risks of extreme dry and wet weather condition derived from the Standardised Precipitation–Evapotranspiration Index (SPEI).

The Geo–referenced Event Dataset from Uppsala Conflict Data Program (UCDP) compiles the fatality figures for each event. We make use of this information to measure the severity of conflict events in different sending countries. Panel A in Fig 3 illustrates the trends in conflict–induced mortality rates. It is evident that the fatality rate in Syria increased dramatically since the outbreak of civil war. The severity peaked in 2014 when over 4 per thousand population were killed, it has then gradually faded. Afghanistan has also experienced a noticeable increase in conflict–induced fatality rate since 2014. The level of fatality, however, is relatively low, compared to that in Syria.

### Climate indicator

To measure climate anomalies, we use the Standardised Precipitation–Evapotranspiration Index (SPEI) with a spatial resolution 0.5° × 0.5° grid and a monthly frequency [58]. SPEI is a normalized indicator for the intensity of extreme climate conditions. A value of +1/-1 indicates a wet/dry condition that is one standard deviation away from the normal condition. The index is considered to be more comprehensive than a single measure of temperature, drought, or rainfall, as it captures the overall balance between precipitation and the sum of evaporation and transpiration.

SPEI has been increasingly used to study the impact of slow onset climate change on international migration. A general finding is that people tend to migrate in response to warmer climate—i.e., migration volume increases as SPEI declines (see e.g., [18, 43]). A recent study further showed that severe drought measured by SPEI played a significant role in driving asylum seeking [13]. An important, but less noticed, shortcoming in these studies is that they do not distinguish between positive (extreme wet) and negative (extreme dry) weather anomalies, but impose a linear relation between SPEI and migration. This implicitly assumes that prospective migrants are only responsive to drought, which could be a strong assumption if a migrant comes from a country frequently exposed to both drought and flood, and may therefore have a two–way response.

Furthermore, the interpretation of the linear relation between SPEI and migration might not be straightforward. For example, an inverse relation between the number of asylum seekers and SPEI (as shown in [13]) would imply that a given change in SPEI from negative to zero (when a dry condition regress to normal) would reduce asylum seeking to the same extent as a given change from zero to positive (when climate deviates from normal to wet). The latter would be counter–intuitive if there is an emergence of wet weather anomaly associated with, for instance, flood or storm.

To test whether people may respond to both dry and wet climate anomalies, we make a distinction between positive and negative value of SPEI. Moreover, to examine the non–linearity between SPEI and migration, we categorize the monthly SPEI data for each 0.5 × 0.5 grid as: extreme wet (if SPEI > 2) and extreme dry (if SPEI < -2). We then aggregate extreme dry and wet incidences over all the grids in a given country during a year. The aggregated number of incidences are then divided by total number of grid–months in each country. We interpret this derived indicator as the annual risk of climate anomalies. These risks are formally defined as,

$$\begin{array}{c} \begin{array}{cc} & S_{o,t}^{Dry}=\frac{\sum_{m=1}^{12}\sum_{g=1}^{G_{o}}I\left(S_{g,m}>2\right)}{G_{o}\times 12}\\ & S_{o,t}^{Wet}=\frac{\sum_{m=1}^{12}\sum_{g=1}^{G_{o}}I\left(S_{g,m}<-2\right)}{G_{o}\times 12} \end{array} \end{array}$$ (1)

where, *I*(*) is an indicator variable equals to one if this grid *g* in a given month *m* is extreme dry (*S* > 2) or extreme wet (*S* < −2). *G*_*o*_ is the total number of grids and *G*_*o*_ × 12 is the grid–months, respectively, for a given origin country *o* during a given year *t*.

Panel C in Fig 3 shows the annual risks of weather anomalies (i.e., the number of anomalies as a percentage share of grid–months in each calendar year). In general, the countries that sent most asylum seekers to the EU are also the ones experienced most frequent climate shocks. Most notably, in 2008, 2010, and 2017, the risks of drought increased substantially in Iraq and Syria. It is also noteworthy that some countries tend to be exposed to both dry and wet anomalies. For example, the risk of extreme wet condition increased to about 8% in 2009, followed by an elevated risk of drought (about 7%) in 2010. The former risk could be an indication of floods, storms, or heavy rainfall, which, however, is often considered as favorable weather conditions (i.e., a large positive value of SPEI) to reduce refugee migration (see e.g., [13]).

### Economic indicator

Income is a key determinant of the attractiveness of each destination. In the economics literature of migration, attractiveness is typically measured by the per capita income difference between the destination and origin (see e.g. [9–12]). Following this, we use World Bank’s annual GDP per capita in constant 2010 US dollar to measure the economic factor driving forced migration. Panel B in Fig 3 depicts the per capita GDP in different countries of origin. While the average income in Syria is not the lowest, it declined substantially during 2011–2014 and remained low thereafter.

## Methodological approaches

The gravity equation in the seminal work of Ravenstein [59] has been the cornerstone for macro analysis of migration flows. In recent decade, economists began to make the macro gravity model consistent with the random utility framework, such that individual migration behavior can be inferred from aggregated flow data. In general, there are two versions of micro-founded gravity model which differ in terms of the distributional assumption of the unobserved component in the random utility function [8]. A restrictive version assumes that the unobserved utility is independent and irrelevant across alternative destinations, i.e., the IIA assumption [60]. This version was first introduced in [9]. Later, a less restrictive gravity model was proposed which relaxed the IIA assumption and is consistent with the nested logistic model [7]. This model generated insights about how migrants’ choice of location may be influenced by the attractiveness of alternative destinations (also known as the multilateral resistance).

In this article, we follow the approach in [7] to derive a less restrictive gravity model, i.e., allowing the attractiveness in one destination to be correlated with other destinations. The probability of an individual to migrate from origin *o* to destination *d* and that of remaining in origin *o* can be respectively written as,

$$\begin{array}{c} \begin{array}{cc} & \text{Pr}\left(M_{o,d,t}\right)=\frac{{\left(e^{V_{o,d,t}}\right)}^{1/\tau }}{e^{V_{o,o,t}}+{\left(e^{V_{o,d,t}}\right)}^{1/\tau }+\sum_{l\in D,l\neq d}e^{V_{o,l,t}}}\\ & \text{Pr}\left(M_{o,o,t}\right)=\frac{e^{V_{o,o,t}}}{e^{V_{o,o,t}}+\sum_{l\in D}e^{V_{o,l,t}}} \end{array} \end{array}$$ (2)

where, *V* is the deterministic utility, *τ* is a dissimilarity parameter with range (0, 1], *D* is a set of alternative destinations.

The dissimilarity parameter *τ* is inversely related to the correlation in the unobserved utility across alternatives [8]. One can think of *τ* as an adjustment of the utility associated with a chosen destination. For example, individuals could be heterogeneous in their preferences for destinations. When a person chooses a destination similar to other alternatives, the correlation in the unobserved utility across alternatives is high and *τ* will be close to zero. The utility gain from the chosen destination (i.e., ${\left(e^{V_{o,d,t}}\right)}^{1/\tau }$) will therefore be adjusted higher by a factor of 1/*τ*. Alternatively, if two potential destinations are saliently different, the dissimilarity parameter *τ* will be close to one and hence equation Eq (2) will reduce to a standard logit model with the IIA assumption.

To link the probabilities of individual migration decisions to macro flow data, we multiply the probabilities in Eq (2) by the population in the origin country, which gives the expected number of people who decide to migrate from *o* to *d* and those who remained in *o*, respectively. Since the population in the origin country is constant across alternatives, the migration rate from *o* to *d* can therefore be simply computed as the ratio of Pr(*M*_*o*,*d*,*t*_) to Pr(*M*_*o*,*o*,*t*_). That is,

$$\begin{array}{c} \begin{array}{cc} E[m_{o,d,t}] & =\frac{\text{Pr}(M_{o,d,t})}{\text{Pr}(M_{o,o,t})}=\frac{{\left(e^{V_{o,d,t}}\right)}^{1/\tau }}{e^{V_{o,o,t}}}\frac{e^{V_{o,o,t}}+\sum_{l\in D}e^{V_{o,l,t}}}{e^{V_{o,o,t}}+{\left(e^{V_{o,d,t}}\right)}^{1/\tau }+\sum_{l\in D,l\neq d}e^{V_{o,l,t}}} \end{array} \end{array}$$ (3)

It is important to note that, as we relax the IIA assumption, the denominators in Eq (2) are no longer the same, and therefore cannot be cancelled out when calculating the migration rate. These denominators are known as the multilateral resistance in [7].

### Empirical gravity models

While it is possible to estimate Eq (3) using some nonlinear optimizers, in practice, Eq (3) is typically linearized by taking logarithm, so the model can be estimated using standard least squares regression.

### Flow fixed-effects models

It is well known that, to obtain unbiased parameter estimates, the residuals in the least square estimator must be uncorrelated with all independent variables. To satisfy this condition, empirical gravity models generally include flow-specific constants to adjust for time-invariant factors, such as colonial ties, common language, geographical and cultural proximity, among others [61, 62]. This approach is commonly known as the (dyad) Flow Fixed-Effects (FE) model (see e.g., [7–12]). However, recent research showed that these conventional FE models are essentially capturing the spatial variation, but not temporal changes, in the panel data on bilateral flows [5]. Hence, this class of model can neither explain, nor predict migration dynamics.

To examine how well the conventional FE gravity models can explain and predict forced migration, we follow the approach in [5] and estimate two versions of Flow Fixed-Effects model. In the first model, we only include flow-specific constants,

$$\text{ln}\;m_{o,d,t}=\alpha_{o,d}+\epsilon_{o,d,t}$$ (4)

where, *α*_*o*,*d*_ is a constant specific to each dyad flow between *o* and *d* (i.e. the flow fixed-effects).

In the second model, we expand Eq 4 by adding time-varying factors, namely the indicators for conflict, climate risks, and economic fluctuations,

$$\begin{array}{c} \begin{array}{cc} \text{ln}\;m_{o,d,t} & =1/\tau V_{o,d,t}-V_{o,o,t}+\alpha_{o,d}+\epsilon_{o,d,t}\\ & V_{o,d,t}=\beta \;\text{ln}\;Y_{d,t}\\ & V_{o,o,t}=\gamma \;\text{ln}\;Y_{o,t-1}-\zeta \;\text{ln}\;D_{o,t-1}-\theta \;\text{ln}\;S_{o,t-1} \end{array} \end{array}$$ (5)

where, *Y*_*d*,*t*_ and *Y*_*o*,*t*−1_ are economic variables in destination and origin, respectively. *D*_*o*,*t*−1_ is a measure of conflicts or geopolitical tensions in origin. *S*_*o*,*t*−1_ are indicators for environmental or climate stressors in origin. *ϵ*_*o*,*d*,*t*_ is an error term which is assumed to resemble the white noise.

By comparing the performance of Eq 5 with that of Eq 4, we can examine to what extent the conventional FE models can capture temporal dynamics of forced migration, on top of the spatial variation that has already been captured by the flow fixed-effects. Specifically, if the simple FE model Eq 4 can perform as good as the model with time-varying predictors Eq 5, then the temporal changes in these predictors are not adding any explanatory or predictive power. This, in turn, implies that Eq 5 can merely describe the spatial patterns of bilateral flows, but cannot explain and predict any temporal dynamics of migration flows [5].

### Flow-specific temporal models

It is important to stress that the effects of predictors in Eq 5 are assumed to be homogeneous. This assumption often leads to large and significant parameter estimates (see e.g., [7, 9–12]). When such an assumption is relaxed, the estimates become highly heterogeneous across contexts (see e.g., [25]). This contrast is, at least partly, because models with homogeneous parameters tend to capture more spatial rather than temporal correlations between migration outcome and predictors [5]. To address this shortcoming, we propose an alternative model,

$$\begin{array}{c} \begin{array}{cc} \text{ln}\;m_{o,d,t} & =1/\tau V_{o,d,t}-V_{o,o,t}+\alpha_{o,d}+\epsilon_{o,d,t}\\ & V_{o,d,t}=\beta_{od}\;\text{ln}\;Y_{d,t}\\ & V_{o,o,t}=\gamma_{od}\;\text{ln}\;Y_{o,t-1}-\zeta_{od}\;\text{ln}\;D_{o,t-1}-\theta_{od}\;\text{ln}\;S_{o,t-1} \end{array} \end{array}$$ (6)

A key difference between Eqs 6 and 5 is that all the parameters are no longer fixed, rather they vary across flows. The model thus becomes less restrictive. Such a parameterization essentially isolates the spatial correlation between predictors and migration outcome. Hence, the parameter estimates will only reflect the relation between temporal dynamics of migration and that of predictors. For this property, we label Eq (6) as “Flow-Specific Temporal Gravity (FTG) model”.

Although less restrictive in parameterization, Eq 6 has an apparent shortcoming—“Data Hungriness”. Compared to the FE model in Eq 5, the number of unknown parameters in Eq 6 increases substantially. To identify these additional flow-specific parameters, the time dimension of panel data needs to be sufficiently long in relation to the number of predictors. This requirement presents a practical challenge, as time-series migration data is generally short. For example, the data used for our empirical analysis below is only 12-year-long. With this short length, the model complexity will need to be constrained, in terms of how many predictors and/or their lags can be included.

The shortcoming discussed above, however, can be tackled by assuming certain probability distribution of the flow-specific parameters. For example, instead of estimating a set of fixed parameter values, Bayesian modelling may provide an effective way to infer the distributions of parameters [5]. This approach could substantially reduce the number of unknown parameters, and hence ease the constrain posed by short time-series data. Nevertheless, for the Bayesian approach to be operational, it is necessary to have some prior knowledge about how the flow-specific parameters might be distributed. However, such prior knowledge is scarce in the context of international migration. In this regard, our FTG model may be useful, as it may generate empirical data on the flow-specific parameters which may serve as priors for future Bayesian inferences.

Another drawback of our proposed FTG model is that, similar to the FE model, many interesting determinants of dyad migration flows (e.g., distance, culture, language, among other proximity) will be vanished into the dyad fixed effects. This issue is not critical in our analysis below, as the objective is to examine the difference in models’ performances (FE vs. FTG), rather than to understand the determinants of forced migration. However, we would like to stress that our FTG model would be less valuable should time-invariant migration drivers be the primary concern.

### Time-varying unobservable factors

As shown in Fig 1, besides the push and pull factors, there are many other factors that may shape people’s migration decisions, but are not observable to researchers—i.e. the “Knowable Unknowns”. For example, migration costs could be an important determinant of where displaced persons choose to move to. Such costs may be in two forms: time–constant and time–varying [8]. Time-constant costs may be influenced by colonial ties, common language, geographical and cultural proximity, among others [61, 62]. These types of costs, however, can be accounted for by the flow-fixed effects.

Time–varying costs, e.g., migration policies, travel expenses, among others, however, are difficult to measure. Price information on travel is not readily available on a global scale, particularly for non–conventional routes that displaced people often travel through. To measure migration policy, the empirical literature generally relies on different visa (waiver) programs (see e.g., [10, 12, 63–65]). However, such policy measures are of little relevance to forced migrants, as they are mostly from low–income countries which tend to have no visa waiver agreements with rich nations, and hence no policy variation overtime.

Furthermore, when deciding where to move, meso–level factors may play an important role. For example, migrant networks are considered to be vital for refugees to access information and to receive financial and practical support [57, 66–69]. Moreover, virtual networks, such as information from social media, are increasingly used by asylum migrants [70]. These channels may potentially reduce migration costs and obstacles, and hence accelerate the process of forced migration. While the importance of migrant networks has been studied to a large extent in qualitative research, they are difficult to analyze quantitatively, as such an analysis would require large–scale data collection in migrants’ countries of origin.

To account for the “Knowable Unknowns” (not only the unobservable factors as discussed above, but also many others that are not mentioned here), we expand Eq 6 by adding a latent state variable Ω_*o*,*d*,*t*_,

$$\begin{array}{c} \begin{array}{cc} \text{ln}\;m_{o,d,t} & =1/\tau V_{o,d,t}-V_{o,o,t}+\alpha_{o,d}+\Omega_{o,d,t}+\epsilon_{o,d,t}\\ & V_{o,d,t}=\beta_{od}\;\text{ln}\;Y_{d,t}\\ & V_{o,o,t}=\gamma_{od}\;\text{ln}\;Y_{o,t-1}-\zeta_{od}\;\text{ln}\;D_{o,t-1}-\theta_{od}\;\text{ln}\;S_{o,t-1} \end{array} \end{array}$$ (7)

In principle, the stochastic process of Ω_*o*,*d*,*t*_ can be inferred with a variety of probability distributions. However, given a short time dimension of our panel data (merely 12 years long), we limit *Ω*_*o*,*d*,*t*_ to follow a simple p order Autoregressive (AR) process. In our estimation, we use the Hyndman–Khandakar algorithm to determine the order of AR based on Akaike Information Criterion—i.e., with the smallest AIC value [71]. With this approach, the process of Ω_*o*,*d*,*t*_ can be written as,

$$\Omega_{o,d,t}=\omega_{o,d}^{1}\Omega_{o,d,t-1}+\omega_{o,d}^{2}\Omega_{o,d,t-2}+\ldots +\omega_{o,d}^{p}\Omega_{o,d,t-p}$$ (8)

where, the Ω′*s* are autoregressive terms with *p* order.

### Model comparison via cross-validation

How well can our FTG models (Eqs 6 and 7) explain and predict migration flows, compared to the FE models (Eqs 4 and 5)? We address this question via a cross-validation procedure, and use Root Mean Squared Error (RMSE) to evaluate models’ performances in the training and testing sets.

As FTG models treat panel data as time-series stratified by dyad flows, the number of parameters thus grows substantially. A consequence of this large parameter set is that, whilst FTG will have a stronger explanatory power than FE in the training set, it might translate into a weaker predictive power (i.e., with a larger testing error). In our cross-validation, we seek to examine the extent to which FTG models are prone to over-fitting. Specifically, we analyze how the testing errors of FTG models may change with respect to the length of time-series. To conduct such an analysis, we apply a cross-validation procedure known as “rolling forecasting origin” [72].

**Fig 4:**
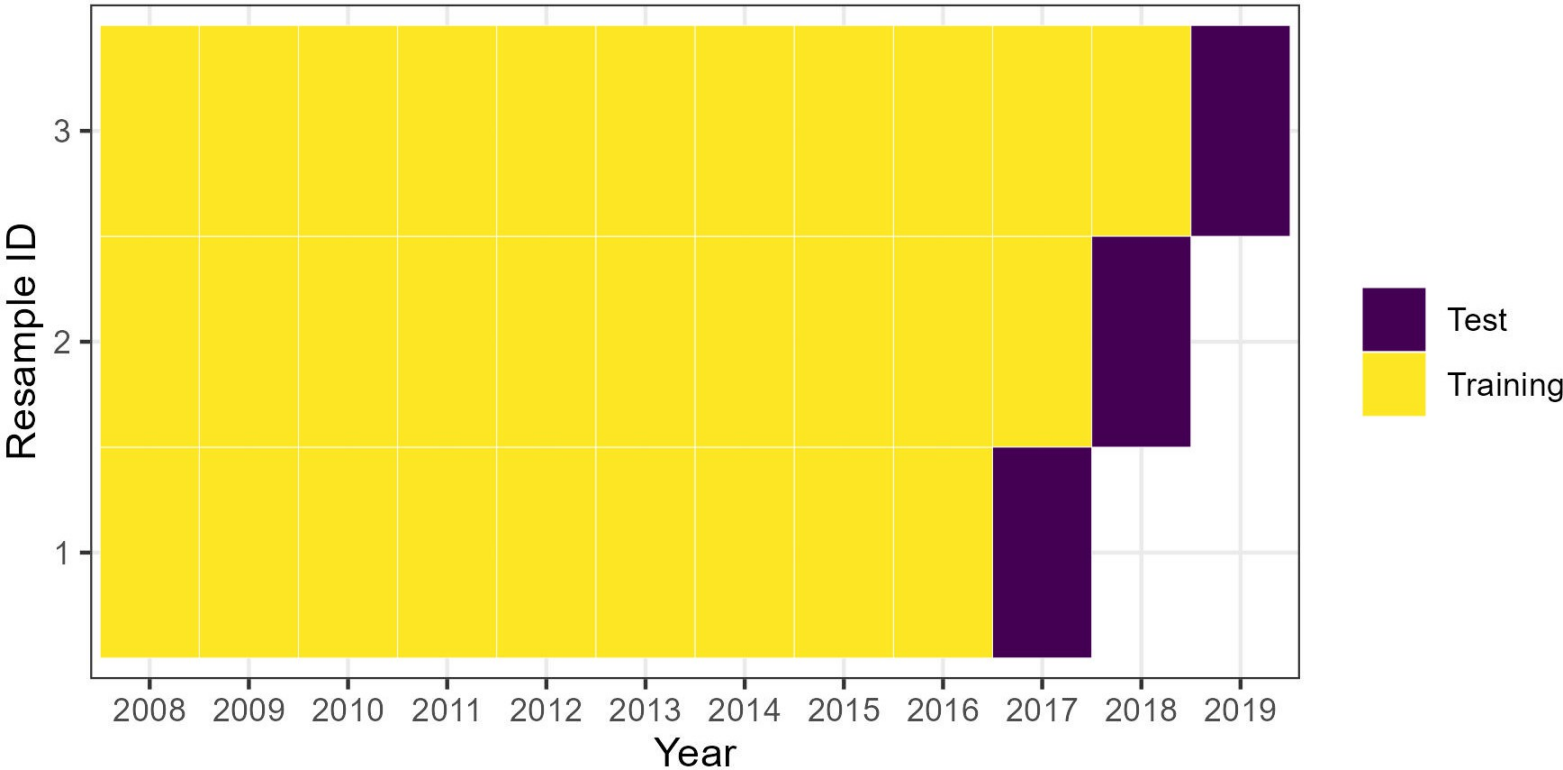
Data splits for cross-validation.

Fig 4 depicts how we split the data into training and testing sets. This cross-validation strategy has two advantages. First, it can help us explore how models’ performances may change with respect to each increment in the length of panel data. Second, the “rolling forecasting origin” approach is also considered to be more appropriate for time-series data [72]. It is important to stress that the choice of this cross-validation method is also to adapt to the flow-specific parameters that are assumed to be fixed in the FE and FTG models. Should these parameters be random draws from certain probability distributions, other cross-validation procedures might be more appropriate, such as K-fold, Leave-One-Out, or Re-sampling with Replacement.

## Results

### Coefficient estimates

**Fig 5:**
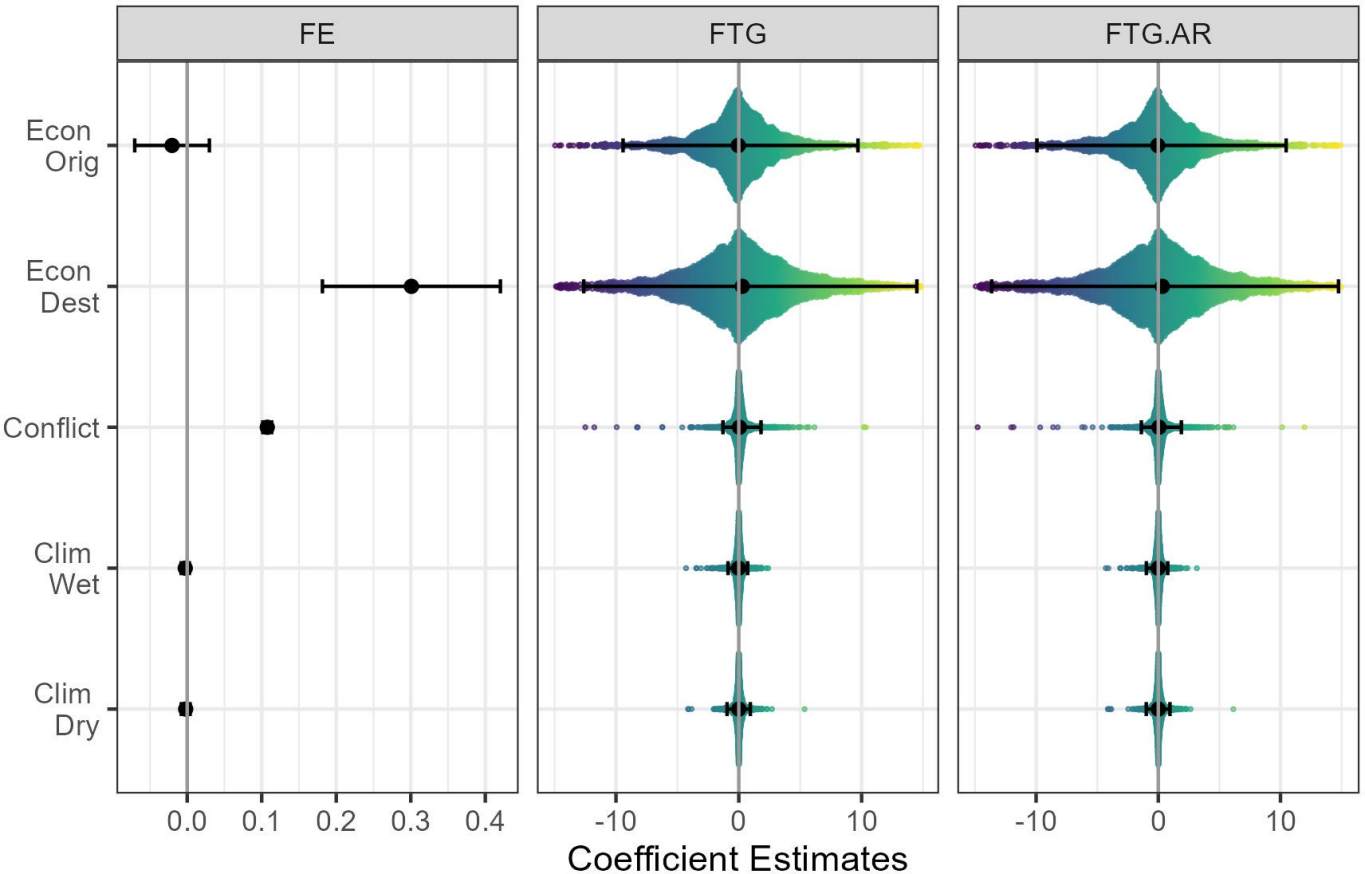
Coefficient estimates by FE and FTG models. Note: The solid bands in FE model represent the 95% confidence intervals of the point estimates, and that in FTG model contain 95% of the estimated flow-specific coefficients.

Fig 5 depicts the coefficient estimates of the time-varying predictors using Eq 5 (left panel), Eq 6 (middle panel), and Eq 7 (right panel). The FE model yields large and significant estimates for conflict and destination economic factors, which are congruent with previous finding (see e.g., [9–12]). These estimates suggest that forced migration to Europe is, on the one hand, pushed by the elevated mortality risks induced by armed conflict, on the other hand, there is a pull effect of higher income in European Economies. The economic push effect (i.e., reduced income in Origin) is insignificant, which contrasts with previous literature. However, it is important to note that, as conflicts or other geopolitical tensions are often accompanied by economic deterioration, the insignificant estimate of per capita GDP in origin could be due to collinearity between the income and conflict indicators.

The estimated climate impact on forced migration is small and insignificant in both FE and FTG models. This finding is in stark contrast to the recent evidence suggesting that droughts in Western Asia played a significant role in driving asylum-related migration to Europe during 2011–2015 [13]. Such a contrasting finding is likely due to different modeling approaches. The econometric model in [13] exploited cross-sectional variation (using pooled bilateral flow data). This approach can easily overestimate the climate impact. For example, Western Asian countries are generally drier than Europe, this spatial climate difference coincides with the large migration flows from Western Asia to the EU. In the FE and FTG models, however, spatial climate variation is captured by the flow fixed-effects. The small estimate could also reflect the reality of migration responses to climate stressors. For example, even in an extreme weather event, the population under pressure might be more likely to be displaced within national borders, rather than move across borders. Furthermore, as climate-induced migration tends to be temporary, those who are initially displaced might eventually return home when conditions return to normal.

The most striking pattern in Fig 3 is that the coefficients estimated by FTG models reveal a pervasive heterogeneity suggesting that the drivers of forced migration are highly dependent on context. Such a pattern is congruent with recent findings which show that about half of the flow-specific parameters are different than the point estimates from dyad fixed-effects models as well as from pooled spatial-temporal models [5, 25]. A key implication of heterogeneous migration responses is that the large and significant point estimates that the conventional FE models typically obtain might not necessarily be valid and consistent (see e.g., [7, 9–12]). In particular, when the effects of regressors are assumed to be homogeneous, FE models could mask the complexity of temporal dynamics that follow very different statistical rules than spatial migration patterns [5]. Indeed, what FTG model demonstrates here is that the drivers of forced migration are non-uniform. This non-uniformity can only be detected when the temporal correlation between predictors and migration outcome are isolated from the spatial correlation.

### Prediction accuracy

**Fig 6:**
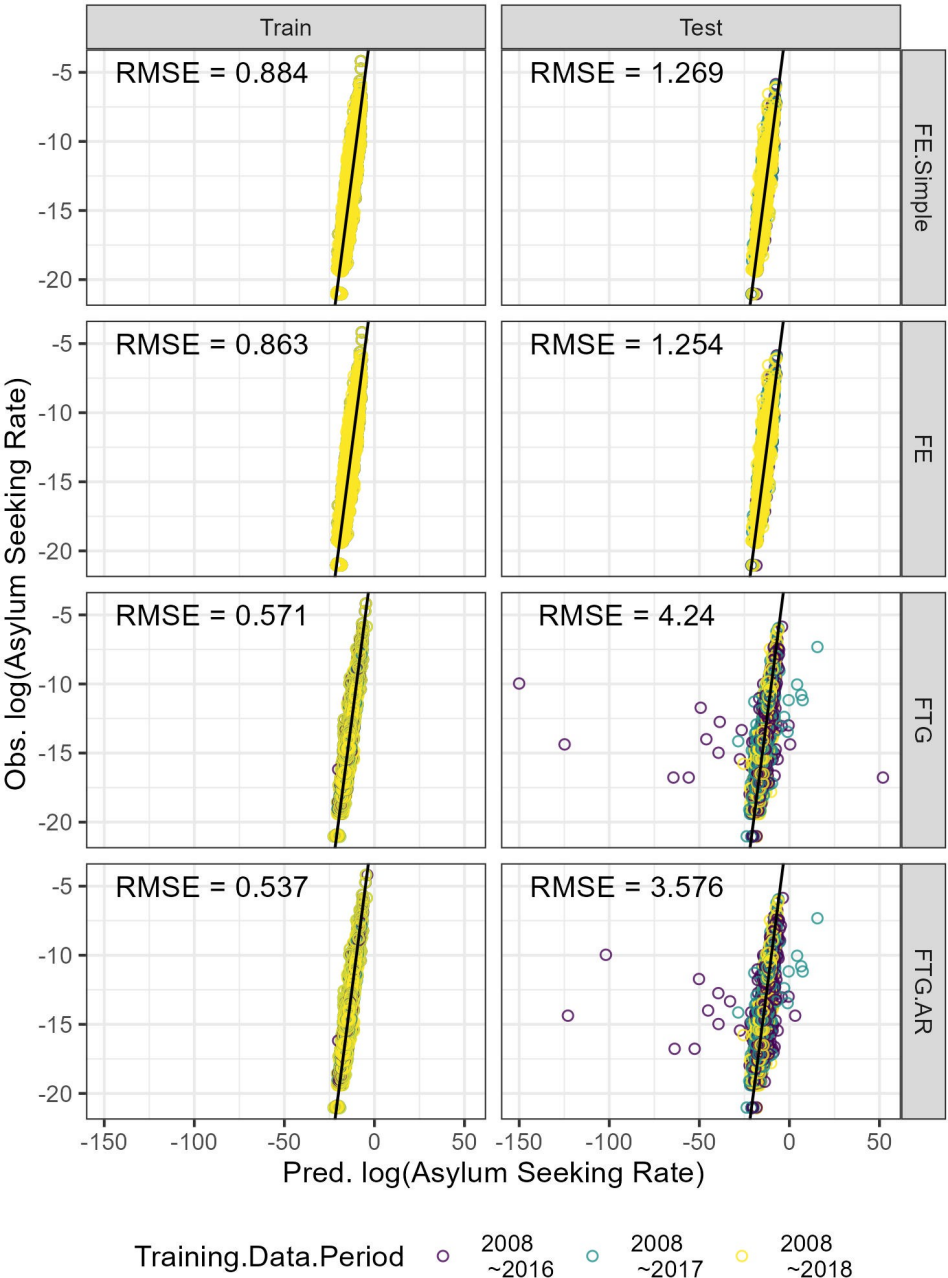
FE and FTG model performance. Each point corresponds to the observed and predicted log ASR for a given flow and in a given year. Points’ colors differentiate the length of training data (as shown in Fig 4). The black solid lines represent 1:1 relationships.

Fig 6 compares the predicted ASR against the actual data. Within the training set, it is clear that FTG models (Eqs 6 and 7) can better explain the spatial-temporal patterns of forced migration, compared to FE models (Eqs 4 and 5); training errors (RMSE) for the former two models are noticeably lower than that for the latter two models. However, in the testing set, FTG models’ prediction accuracy is substantially worse (the testing errors for FTG models are much larger than that for FE models). This indicates that, when models treat panel data as flow-specific time-series, they are prone to over-fitting. However, it is noteworthy that the extent to which FTG models over-fit is not constant, rather it varies depending on how long the time dimension of training data is. To inspect this variation, we compute the training and testing errors by data splits.

**Fig 7:**
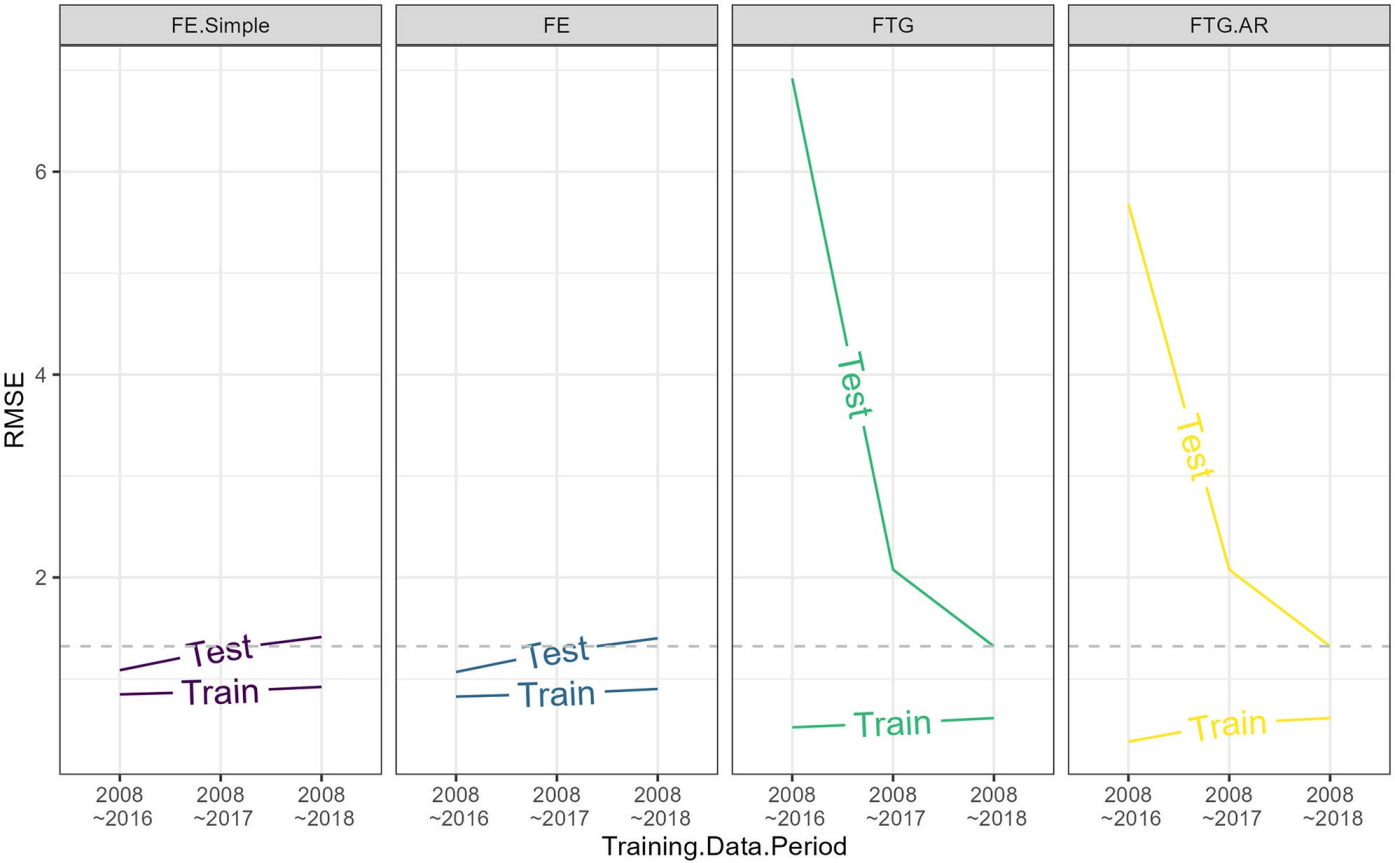
Effect of panel data length on model performance. Changes in training and testing errors (RMSE) with respect to the length of training data (as shown in Fig 4).

Fig 7 depicts how the training and testing RMSE evolve over different lengths of training sets. It is clear that when the training data is relatively short in length, the testing performance of FTG models is extremely poor. However, as the training set increases in length, FTG models’ predictions become increasingly accurate. Most notably, when models are trained on 11 years of data (i.e., 2008–2018), the testing RMSE for FTG models become smaller than that for the FE models.

The most striking finding from Fig 7 is that, as the panel data becomes longer in its time dimension, the discrepancies between training and testing errors tend to diverge for FE models, whereas that for FTG models tend to converge. These contrasting patterns reveal a salient difference between these two classes of model, namely, when training data expands over the time dimension, the chance of over-fitting will increase for FE models, whereas that for FTG models will decrease. A key implication of this difference is that FTG models are “Data Hungry”. Specifically, to be capable of making reliable inferences and near-term predictions, FTG models need to be trained on flow-specific time-series that is sufficiently long relative to the number of predictors.

Given the predominant role that the flow fixed-effects model has played in the field of modelling and forecasting migration flows, it is critical to understand why its performance worsens when the panel data grow in length. A plausible explanation is that the FE model can capture only spatial, but not temporal, variation in migration flows [5]. This can be seen by comparing the simple FE model (Eq 4) with the full-scale FE model (Eq 5) in Fig 7; the performances of the two FE models are identical, suggesting that it is the flow-specific constants, but not the time-varying predictors in Eq 5, capturing all the variation in migration flows. Given such a model behavior, it is not difficult to see that the FE model will become less predictive if the time dimension of panel data expands, as the spatial variation captured by the flow constants will become less dominant when the time-series flow data lengthens.

**Fig 8:**
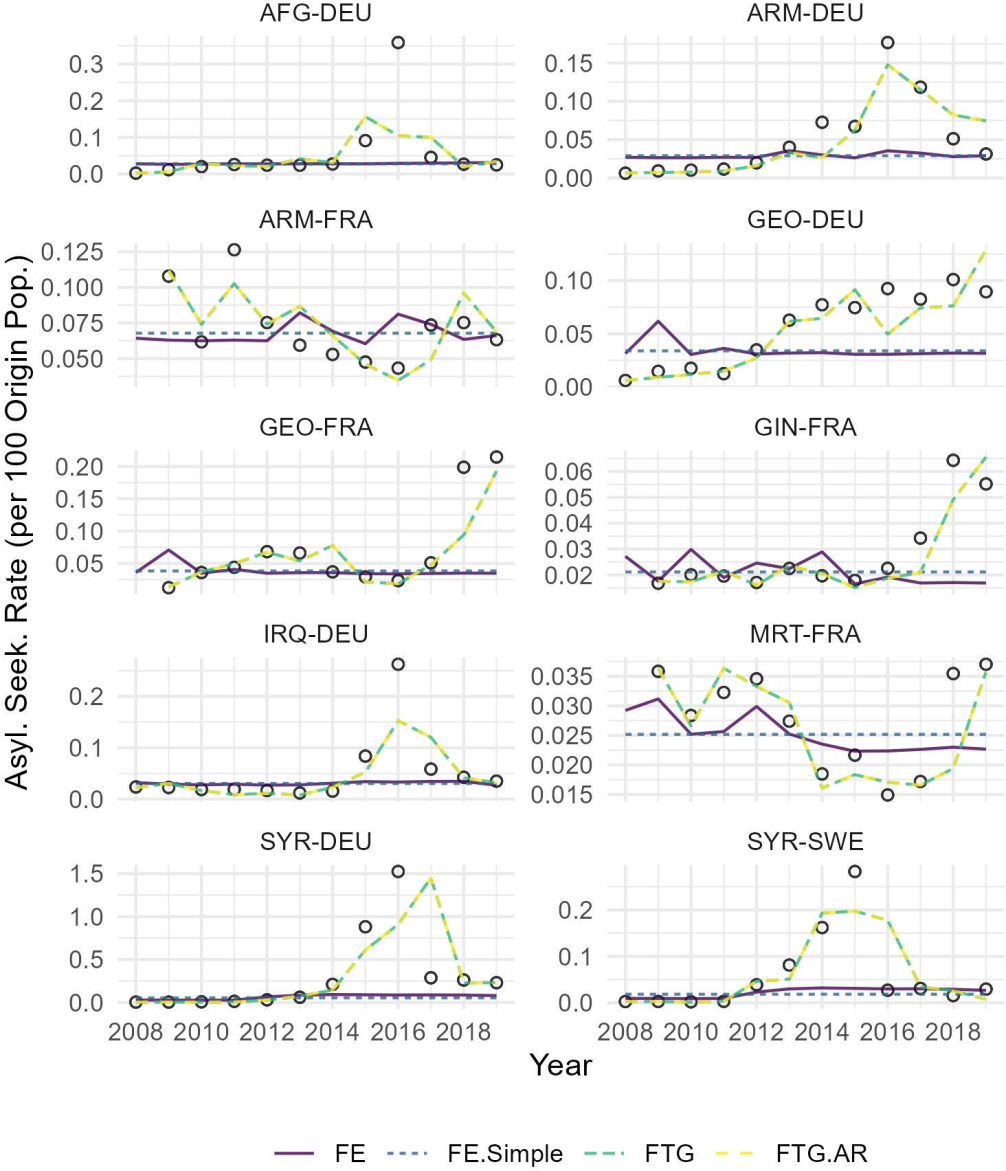
Observed and predicted temporal trends of forced migration. The observed data are represented by dots. The selected flows represent the top 10 asylum-related migration flows to Europe during 2008-2019. The temporal trends are predicted by the model trained on the data 2008-2018 (i.e., the longest data split in Fig 4).

The stochastic component, namely the autoregressive (AR) term in Eq 7, does not seem to add any predictive power. Specifically, the testing performances of the two FTG models (with and without an AR component) in Fig 7 appear to be nearly the same, with an exception of when the training data is short in time dimension (9-year-long). Moreover, the temporal trends predicted by the two FTG models are roughly identical (see Fig 8). Nevertheless, these results need to be interpreted with caution. Specifically, they do not necessarily mean that there is no stochasticity in the temporal dynamics of forced migration. Rather, they imply that the assumed AR process may be too restrictive to represent the temporal dynamics of unobserved factors.

Overall, the model comparisons presented above suggest that FTG models are preferable for two key reasons. First, this class of model can account for heterogeneity in migration responses to time-varying factors, which is pervasive (see Fig 5). Second, the predictions of FTG models, compared to that of FE models, are more accurate in both training and testing sets (see Fig 7). They can also better resemble the observed temporal trends (see Fig 8).

However, it is important to note that the performances of FTG models are highly dependent on the length of flow time-series data; the data needs to be sufficiently long relative to the number of parameters, in order for FTG models to make reliable inferences and predictions. Another important point is that, despite their better performances relative to FE models, FTG models are far from being perfect. As can be seen from Fig 8, for some flows, there are growing discrepancies between FTG models’ predictions and observed data overtime, particularly after 2015, suggesting that the innovations in the AR component have an unstable variance. While this issue is difficult to address in this article due to limited length in our time-series data, it can and should be investigated in the future when longer flow-specific time-series data become available. By then, the performance of FTG models can be potentially enhanced by including more time-varying predictors, and/or by using more flexible probability distributions to model the processes of the latent state variable.

## Conclusions

As spatial-temporal panel data on human migration become increasingly available, the techniques to fit gravity models have evolved significantly during the last decade, from simple cross-sectional analysis of spatial patterns to more advanced Fixed-Effects (FE) models. The latter model class is designed to infer migration responses to not only spatial, but also temporal variations in economic, geopolitical, environmental factors, among others. However, a recent study demonstrated that such approach failed to explain, and hence to predict, temporal dynamics, it can merely describe spatial patterns of international migration [5].

In this article, we derived a Flow–Specific Temporal Gravity (FTG) model that is theoretically informed by the random utility framework. Using EUROSTAT asylum statistics together with climate, conflict, and economic indicators, we demonstrated how the FTG model can account for heterogeneous migration behavior. We also assessed and compared models’ performances at various lengths of training data. The results suggest that, as flow time-series data lengthens, FTG models’ predictions can be increasingly accurate, whereas FE models become less predictive. However, we would like to highlight a key limitation in our analysis, and discussing how our results might depend on this limitation.

The empirical gravity models specified in our analysis (Eqs 5, 6 and 7) are adapted to the length of flow time-series. With merely 12-year-long data for each flow, FTG models are constrained by how many predictors can be included. A key implication of this constrain is that such an approach may be less valuable if the research interest goes beyond the migration responses to economic, conflict and climate factors. In other words, FE models may still be attractive if a broader set of explanatory variables are of interest, e.g., migrant networks, policies, and migration costs, among others. In a similar vein, FE models can also accommodate more lagged predictors which may be important for understanding the temporality of forced migration. Nevertheless, a larger set of predictors might not necessarily imply a greater predictive power of FE models. This is because the performances of the two FE models examined above (with or without time-varying predictors) are identical (see Fig 7). Hence, in the context of predictive analysis, adding more time-varying predictors is unlikely to enhance FE models’ performances.

“Data Hungriness” is an apparent shortcoming of FTG models. As the length of time-series flow data are generally limited, building more complex FTG models is therefore challenging. To conclude, we would like to discuss a few options that might potentially ease such a challenge.

While it takes time for migration data to grow in length, increasing the frequency of analysis may permit FTG models to include a richer set of predictors and/or their lags. For example, weekly asylum statistics are now being compiled by EUROSTAT which have already been used to model refugee migration to Europe [4]. However, even though high frequency data is accessible, certain regularization methods are still needed. For example, in [4], Elastic-Net was applied to remove redundant predictors in the time-series model. Moreover, when using high frequency data, the analysis will need to exclude economic incentives—a central focus in the random utility framework. This is because economic indicators, such as GDP, are mostly available annually. With this in mind, a fruitful research avenue could be to explore alternative data sources for measuring economic conditions in both sending and receiving countries.

Another option to ease the constraints posed by short time-series could be to appropriately group the data, while keeping it at a low frequency [5]. For example, the flow time-series can be grouped by countries of origin, which will lead to smaller origin-specific panels. This setup can be useful for analyzing heterogeneous migration behavior across origins. However, an implicit assumption here is that all the same-origin migrants are of similar characteristics regardless of which destination they choose. If such an assumption does not hold (e.g., those who choose to migrate to Sweden are dissimilar to those who move to France), then the analysis would mask important heterogeneity in migration responses to time-varying pull factors.

Finally, given that the distributions of flow-specific parameter estimates (as shown in Fig 5) have approximately symmetric and skewed shapes, a natural extension of FTG models would be to represent the parameters with probability distributions. As suggested by [5], instead of estimating a set of fixed parameter values, Bayesian modelling may provide an effective way to infer the distributions of parameters. This approach could substantially reduce the number of model parameters to be estimated, and hence ease the constraints posed by short time-series data. It could also be a fruitful approach for modelling the stochasticity of latent state variables (such as Ω_*o*,*d*,*t*_ in Eq 7).
